# Cholinesterase inhibitory, anti-amyloidogenic and neuroprotective effect of the medicinal plant *Grewia tiliaefolia* – An *in vitro* and *in silico* study

**DOI:** 10.1080/13880209.2016.1241811

**Published:** 2016-12-08

**Authors:** Dicson Sheeja Malar, Rajamohamed Beema Shafreen, Shunmugiah Karutha Pandian, Kasi Pandima Devi

**Affiliations:** a Department of Biotechnology, Alagappa University, Karaikudi, Tamil Nadu, India;; b Centre for Nanoscience and Nanotechnology, Sathyabama University, Chennai, India

**Keywords:** Antioxidant, docking, Alzheimer’s disease, neurotoxicity, aggregation

## Abstract

**Context:***Grewia tiliaefolia* Vahl. (Tiliaceae) is a sub-tropical plant used as an indigenous medicine in India. However, its efficacy has not been evaluated against Alzheimer’s disease.

**Objectives:** The objective of this study is to evaluate cholinesterase inhibitory, anti-aggregation and neuroprotective activity of *G. tiliaefolia*.

**Materials and method:***Grewia tiliaefolia* leaves were collected from Eastern Ghats region, India, and subjected to successive extraction (petroleum ether, chloroform, ethyl acetate, methanol and water). The extracts were subjected to *in vitro* antioxidant, anticholinesterase and anti-aggregation assays. The active methanol extract (MEGT) was separated using column chromatography. LC-MS analysis was done and the obtained compounds were docked against acetylcholinesterase (AChE) enzyme to identify the active component.

**Results:** Antioxidant assays demonstrated that the MEGT showed significant free radical scavenging activity at the IC_50_ value of 71.5 ± 1.12 μg/mL. MEGT also exhibited significant dual cholinesterase inhibition with IC_50_ value of 64.26 ± 2.56 and 54 ± 0.7 μg/mL for acetyl and butyrylcholinesterase (BChE), respectively. Also, MEGT showed significant anti-aggregation activity by preventing the oligomerization of Aβ_25–35_. Further, MEGT increased the viability of Neuro2a cells up to 95% against Aβ_25-35_ neurotoxicity. LC-MS analysis revealed the presence of 16 compounds including vitexin, ellagic acid, isovitexin, etc. *In silico* analysis revealed that vitexin binds effectively with AChE through strong hydrogen bonding. These results were further confirmed by evaluating the activity of vitexin *in vitro,* which showed dual cholinesterase inhibition with IC_50_ value of 15.21 ± 0.41 and 19.75 ± 0.16 μM for acetyl and butyrlcholinesterase, respectively.

**Discussion and conclusion:***Grewia tiliaefolia* can be considered as a promising therapeutic agent for the treatment of AD.

## Introduction

Alzheimer’s disease (AD), a progressive neurodegenerative disorder affecting the ageing population, is characterized by progressive loss of memory and deterioration of intellectual functions, thereby affecting activities of daily life (Kandale et al. 2013). According to World Alzheimer Report, approximately 46 million people were reported to be affected by dementia in 2015, and it is expected that this figure may increase up to 131.5 million by the end of 2050, of which AD accounts for around 80% of cases (Matrone & Brattico 2015; World Alzheimer’s Report, 2015). Multiple molecular mechanisms such as abnormal deposition of amyloid plaques and tau protein aggregation, excessive metal ions, oxidative stress, slow inflammatory process and reduced acetylcholine level were thought to be the main reasons involved behind the pathogenesis of this disease (Mohandas et al. 2009). There is a considerable amount of drug research that is currently taking place to discover effective therapeutic treatments for AD. A range of molecules targeting amyloid beta (Aβ) aggregation, β, γ-secretases, which are involved in amyloid precursor protein (APP) processing, and modulators of tau phosphorylation are under various phases of clinical trials (Schneider et al. 2014). In spite of these clinical strategies, cholinesterase inhibitors (ChEI’s) are the first pharmacological treatments for AD to be approved by the US Food and Drug Administration (FDA). Acetylcholinesterase (AChE) and butyrylcholinesterase (BChE) enzymes play an important role in the pathophysiology of AD by hydrolyzing the neurotransmitter acetylcholine (ACh) thereby disrupting the synaptic transmission (Hebert et al. 1995). ChEI’s enhance the central cholinergic function by inhibiting the enzymes that degrade ACh, thereby increasing the availability of ACh to stimulate nicotinic and muscarinic receptors within the brain (Colović et al. 2013). Since their introduction into clinical practice, ChEI’s have been, and remain, the standard approach to the symptomatic treatment of AD. Large-scale placebo-controlled trials of synthetic AD drugs like tacrine, donepezil, rivastigmine and galantamine have shown modest symptomatic benefits in patients with mild to moderate AD (Corey-Bloom et al. 1998; Knapp et al. 1994; Mintzer & Kershaw 2003; Boada-Rovira et al. 2004). But mostly these drugs act on the basis of one target strategy, and when patients no longer take the drug, their symptom of AD returned and also these drugs have shown adverse side effects.

Besides the role of cholinesterase enzyme in the pathology of AD, synthesis, aggregation and deposition of Aβ play a major role in cognitive impairment. The deposition of Aβ in the brain causes severe stress to the neuronal cells leading to their death (Prasansuklab & Tencomnao 2013). The growing evidence of oxidative stress-mediated damages to the DNA, proteins and lipids during AD has prompted for the identification of new molecules that not only serve as cholinesterase inhibitors but also play a vital role in scavenging the free radicals formed during the pathogenesis of the disease.

Plants with medicinal values are the resource for simple to complex secondary metabolites with impending therapeutic applications. These metabolites are effectively known to prevent several chronic diseases including AD, through different mechanisms like prevention of oxidative stress, inhibition or modulation of enzymes and receptor, interfering with the cellular signals, and so on. A wide variety of plants have been explored for their pharmacological properties against AD upon the detection of the beneficial roles of the plant-derived compounds like huperzine A, galantamine and physostigmine (Shaw et al. 1985; Ashani et al. 1992; Coyle & Kershaw 2001).

*Grewia* genus, comprising around 150 species, belongs to the family Tiliaceae and is distributed widely in tropical and sub-tropical regions (Ullah et al. 2012). Approximately 40 species of this genus are reported to be present in India (Hiwale 2015), many of which are having medicinal properties; one among them is *Grewia tiliaefolia*. This plant has been used as astringent, expectorant, antipruritic and aphrodisiac by the traditional medicinal practitioners and local tribes in India (Selvam et al. 2010). Several works in this plant have revealed its antioxidant, anticancer and hepatoprotective activities both under *in vitro* and *in vivo* condition. Badami et al. (2003) reported that the lupeol isolated from the stem bark of *G. tiliaefolia* shows cytotoxic activity against Vero, Hep-2 and B_16_F_10_ cell lines. Selvam et al. (2010) reported that the methanol extract of bark has potent antiproliferative activity against MCF7, A549 and HepG-2 cell lines. Two γ-lactones, D-erythro 2-hexenoic acid γ-lactone and gulonic acid γ-lactone isolated from the stem bark of *G. tiliaefolia* have shown potent hepatoprotective activity in CCl_4_-intoxicated rats (Khadeer-Ahamed et al. 2010). Leaves of this plant are reported to be consumed as vegetables (Patil & Patil 2006). Being used in Ayurvedic medicine in India, this plant has not been previously explored for its neuroprotective effect. The current study is performed to investigate the cholinesterase inhibitory, anti-aggregation and neuroprotective effect of *G. tiliaefolia* and also to reveal the possible interaction of the active components identified with AChE through docking studies.

## Materials and methods

### Chemicals and reagents

2,2-Dephenyl-1-picrylhydrazyl (DPPH), AChE enzyme and vitexin were purchased from Sigma Aldrich, St. Louis, MO. BChE enzyme was procured from MP Biomedicals, Santa Ana, CA. Sodium nitroprusside, butylated hydroxytoluene (BHT), 2,4,6-tripyridyl-s-triazine (TPTZ), acetylthiocholine iodide (ATCI), butyrylthiocholine iodide (BTCI), 5,5′-dithiobis(2-nitrobenzoic acid) (DTNB), Dulbecco’s Modified Eagle Medium (DMEM) and fetal bovine serum (FBS) were obtained from Himedia Laboratories, Mumbai, India. Solvents were procured from Sisco Research Laboratories, Mumbai, India. All other chemicals and solvents used in the experiments are of high grade and purity.

### Plant collection and extract preparation

*Grewia tiliaefolia* was collected from Sirumalai Hills (Eastern Ghats, 10°11'39.3''N and 77°59′48.0″E) of India in March 2012, and authenticated by Dr. S. John Britto, Director, The Rapinet Herbarium and Centre for Molecular Systematics, St. Joseph’s College, Tiruchirapalli, Tamil Nadu, India, and the voucher specimen was deposited at Department of Biotechnology, Alagappa University, under the accession no. DSM001. Leaves were separated, washed, shade dried, powdered and subjected to successive extraction using Soxhlet apparatus with solvents of varying polarities ranging from non-polar to polar (petroleum ether, chloroform, ethyl acetate, methanol and water). The extracts were collected and dried using vacuum rotary evaporator until no traces of solvents are present and stored at −20 °C until use. The yield of the extract is calculated as follows:

$$\mathrm{Yield}=\frac{\mathrm{Amount}\;\mathrm{of}\;\mathrm{extract}\;\mathrm{obtained}}{\mathrm{Amount}\;\mathrm{of}\;\mathrm{leaf}\;\mathrm{packed}\;\mathrm{for}\;\mathrm{extraction}}$$

The dried extract was dissolved in 0.01% Tween 20 (non-polar extracts) or distilled water (polar extracts) before the start of the experiment.

### DPPH radical scavenging activity

DPPH radical scavenging activity of the leaf extracts of *G. tiliaefolia* was measured using the method developed by Blois (1958). DPPH (0.1 mM) was added with various solvent extracts of different concentrations (100–500 μg/mL) and the reaction mixture was allowed to react in the dark for 30 min. The absorbance was measured at 517 nm. BHT (20–100 μg/mL) was used as the standard. The percentage of DPPH radical scavenging activity was determined as follows:

$$\mathrm{Percentage}\;\mathrm{of}\;\mathrm{inhibition}=\frac{{\mathrm{Absorbance}}_{\mathrm{Control}}-\mathrm{Absorban}ce_{\mathrm{Test}}}{{\mathrm{Absorbance}}_{\mathrm{Control}}}$$

### Nitric oxide radical scavenging activity

Nitric oxide scavenging activity of *G. tiliaefolia* extracts were determined by Griess reaction (Garrat 1964). The reaction mixture containing 10 mM sodium nitroprusside in phosphate-buffered saline (pH 7.4) and plant extracts (100–500 μg/mL) were incubated at 25 °C for 150 min. To this reaction mixture, Griess reagent (1% sulphanilamide in 5% orthophosphoric acid and 0.1% naphthyl ethylenediamine in distilled water) was added. The solution was mixed and allowed to stand for 10 min at 25 °C and the absorbance was measured at 546 nm. BHT (50–250 μg/mL) was used as the standard.

### Ferric reducing antioxidant power assay

The ability of the plant extracts to reduce the Fe(III)-TPTZ (2,4,6-tris(2-pyridyl)-s-triazine) complex to Fe(II)-TPTZ was determined according to the method of Benzie and Strain (1996). To 50 μL of various solvent extracts, 150 μL of water and 1.5 mL freshly prepared FRAP reagent (0.3 M acetate buffer (pH 3.6), 10 mM TPTZ in 40 mM HCl and 20 mM FeCl_3_ in the ratio 10:1:1) were added, and the absorbance was measured for 4 min at 593 nm. The relative activity of the extracts was compared with standard ascorbic acid (20–100 μg/mL).

### Reducing power assay

Reducing power of the plant extracts was measured using the method of Oyaizu (1986). To the various plant extracts (100–500 μg/mL), 1 mL of 0.2 M phosphate buffer (pH 6.6) and 1 mL of 1% potassium ferricyanide was added and incubated at 50 °C for 20 min. To this mixture, 1 mL of 10% TCA was added and centrifuged at 3000 rpm for 10 min. To 1 mL of the supernatant, 1 mL of H_2_O and 0.5 mL of 0.1% FeCl_3_ were added and the absorbance was measured at 700 nm. Ascorbic acid (20–100 μg/mL) was used as the standard.

### Estimation of total polyphenols

Total polyphenolics of various solvent extracts were spectrophotometrically quantified using gallic acid as the standard (Singleton & Rossi 1965). The extract (100 μL) was incubated with 1 mL of Folin–Ciocalteu reagent at room temperature for 5 min. Sodium carbonate (7%; 1 mL) was added to the reaction mixture and kept under incubation for 90 min and the reading was taken at 750 nm. The total phenolic is expressed as gallic acid equivalent (GAE) in milligrams per gram of dry sample.

### Cholinesterase inhibitory assay

AChE and BChE inhibitory activity for various solvent extracts was measured using the method of Ellman et al. (1961). Initially, 10 μL of 0.1 U enzyme (AChE or BChE) and 100–500 μg/mL of the extracts were incubated and allowed to react for 45 min in a 96-well plate. The reaction was stopped by the addition of 50 mM Tris-HCl pH 8.0 for AChE and pH 7.4 for BChE. To this mixture, 125 μL of 3 mM DTNB and 50 μL of 15 mM substrate (ATCI or BTCI) were added to initiate the reaction. The absorbance was taken at 405 nm and the % inhibition was calculated.

### Analysis of anti-aggregation property by thioflavin-T assay

Aβ_25–35_ peptide was first solubilized in hexafluoro-2-propanol to form monomers and dried under vacuum. The dried powder was dissolved in MilliQ before use. Freshly prepared Aβ_25–35_ (100 μM) monomer was incubated in Tris-HCl buffer pH 7.4 at 37 °C for 20 h to form oligomers. To the oligomer mixture, 50 μg/mL methanol extract of *G. tiliaefolia* was added and subjected to incubation for 48 h (the optimum dose was fixed based on preliminary experiments; data not shown). Aliquots were drawn from the incubation mixture at 0, 20 and 48 h, respectively, for thioflavin T (Th-T) fluorimetric assay. The solutions containing Aβ_25–35_ with/without extract (0, 20 and 48 h) was added to 5 μM Th-T and the reaction volume was made to 1 mL with 50 mM glycine–NaOH buffer (pH 8.5), and fluorescence intensities were measured at 450 nm (excitation) and 485 nm (emission) under time-resolved fluorescence mode in spectrofluorimeter (Molecular Device Spectramax M3, Molecular Device, Sunnyvale, CA) (Syad & Devi 2015). The background Th-T fluorescence intensity was subtracted from the experimental values. Galantamine (50 μM) was used as a positive control.

### Confocal microscopic analysis

About 2.5 μL aliquot of the samples (0, 20 and 48 h) was diluted 2× with 5 μM Th-T in 50 mM glycine–NaOH buffer (pH 8.5) and transferred onto a slide. Fluorescent signals (488 nm) were then visualized by confocal laser microscope (CLSM FV300, Olympus, Tokyo, Japan).

### Determination of anti-aggregation property by FTIR analysis

The alteration in the chemical structure of Aβ_25–35_ upon treatment with MEGT was recorded in the absorbance mode using Nicolet iS5 FTIR spectrometer (Thermo Fischer Scientific Inc, Waltham, MA) according to the protocol described by Shanmugam and Jayakumar (2004). About 10 μL of the samples were mixed with KBr crystals and lyophilized to remove the moisture content and then pelleted. The pellet was then mounted on the pellet holder for spectral analysis. The spectra were taken in the wavelength range of 400–4000 cm^−1^ at a spectral resolution of 4 cm^−1^.

### MTT assay

Neuro2a cells were purchased from National Centre for Cell Sciences (NCCS), India. Cells were cultured with DMEM medium supplemented with 10% FBS and 1× penicillin–streptomycin antibiotic and maintained at 37 °C in a humidified incubator containing 5% CO_2_. Neuro2a cells (2 × 10^5^ cells) were pretreated with the methanol extract of *G. tiliaefolia* (25–100 μg/mL) for 2 h and then treated with 50 μM Aβ_25–35_ for 24 h. After treatment, cells were washed with PBS and then with MTT (1 mg/mL) and incubated for 3 h at 37 °C. After 3 h, MTT was removed and the formed crystals were solubilized by the addition of dimethyl sulphoxide and the absorbance was measured at 540 nm using spectrophotometer (Molecular device Spectramax M3, equipped with Softmax Pro V 5.4.1 software, Molecular Device, Sunnyvale, CA) (Suganthy & Devi 2016).

### Bioactive guided fractionation using column chromatography

The dried methanol extract of *G. tiliaefolia* leaf (5 g) was subjected to column chromatography (silica gel 60–120 mesh) and eluted with various solvents of increasing polarity. Fractions were collected, dried and subjected to DPPH assay and cholinesterase inhibitory assay as described earlier.

### Identification of compounds through LC-MS analysis

The active fraction was subjected to LC-MS analysis using reverse phase C-18 column. Both positive and negative modes of electron spray ionization were carried out in the *m*/*z* range of 50–1000 for the detection of compounds in the fraction. The mobile phase used for the separation was a mixture of water:methanol:THF (50:40:10). The flow rate was maintained as 1.5 mL/min. The compounds were identified by comparing their *m*/*z* ratio with those on the stored library (Metwin version 2.0, Molecular Device, Sunnyvale, CA).

### Molecular docking

The chemical compounds revealed through LC-MS analysis were further subjected to molecular docking. Donepezil, huperzine A, galantamine and tacrine were used as the positive control. Three-dimensional (3D) structures of all the identified compounds were retrieved from the Pubchem database (http://pubchem.ncbi.nlm.nih.gov). The compounds were docked into the rigid binding pocket of AChE. The 3D structure of AChE was downloaded from Protein Data Bank (PDB code: 1B41) and molecular docking studies were performed using Ligand Fit module available in the Discovery studio package, version 2.5 (Accelrys, Inc., San Diego, CA). The binding pocket of the AChE protein was identified using "Define and edit binding site" protocol available from Discovery Studio. The presence of the water molecules in the AChE was removed and was further subjected to docking analysis for the assortment of compound with promising AChE inhibitory activity.

### Estimation of antioxidant and cholinesterase inhibitory effect of vitexin

Antioxidant and cholinesterase inhibitory effect of vitexin (20–100 μM) was evaluated according to the protocols described earlier with the respective standards of the same concentration.

### HPTLC analysis

Automated application of standard vitexin, methanol extract and F-12 was applied on silica gel TLC plates (60F-254) with Linomat sample applicator using the HPTLC system (CAMAG, Muttenz, Switzerland). Ethyl acetate:formic acid:water (9:1:1) was used as the mobile phase in the CAMAG twin trough glass chamber under saturated conditions, and the plate was documented under 254 and 366 nm using CAMAG Reprostar 3 (CAMAG, Muttenz, Switzerland). For imaging, the plate was scanned densitometrically at 335 nm using TLC Scanner 3 (absorbance mode).

### Statistical analysis

All the experiments were conducted in triplicate and one-way ANOVA (SPSS 17, SPSS Inc., Chicago, IL) was used to compare the mean values of each treatment. Significant differences between the means of parameters were determined by using Duncan’s test (*p* < 0.05) comparing between the groups control and treated. IC_50_ values were calculated using Probit software (Probit Software Ltd., Morton Grove, IL).

## Result

### Antioxidant assays

**Figure 1.**
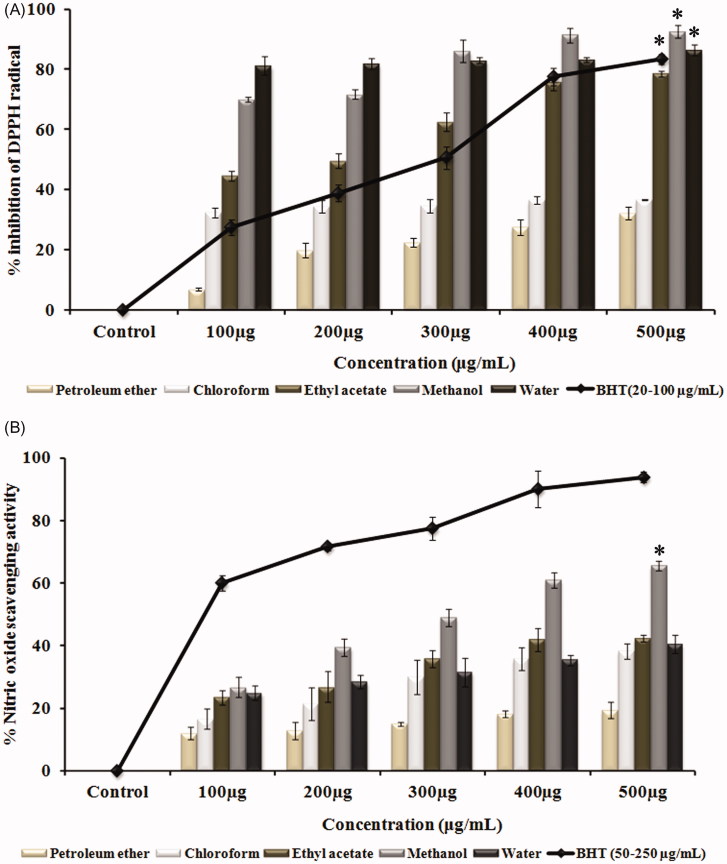
(A) Free radical scavenging activity of various solvent extracts of *G. tiliaefolia* (100–500 μg/mL) in comparison with standard BHT (20–100 μg/mL). (B) Nitric oxide scavenging activity of various solvent extracts of *G. tiliaefolia* (100–500 μg/mL) and standard BHT (50–250 μg/mL). *Significant level at *p* < 0.05 (control versus treated).

DPPH assay and nitric oxide scavenging assay, which provides simplified version to detect the antioxidant properties of various solvent extracts, shows that the scavenging of free radicals by the extracts occurred in a dose-dependent manner (Figure 1(A)). Methanol extract at 500 μg/mL concentration (92%) has shown significant free radical scavenging activity (*p <* 0.05) with an IC_50_ value of 71.5 ± 1.12 μg/mL when compared with the control. Also, the methanol extract of *G. tiliaefolia* at an IC_50_ value of 304.85 ± 9.06 μg/mL was able to inhibit the production of nitric oxide implicating the nitric oxide scavenging ability and thereby impeding the adverse effects created due to its overproduction (Figure 1(B)).

**Figure 2.**
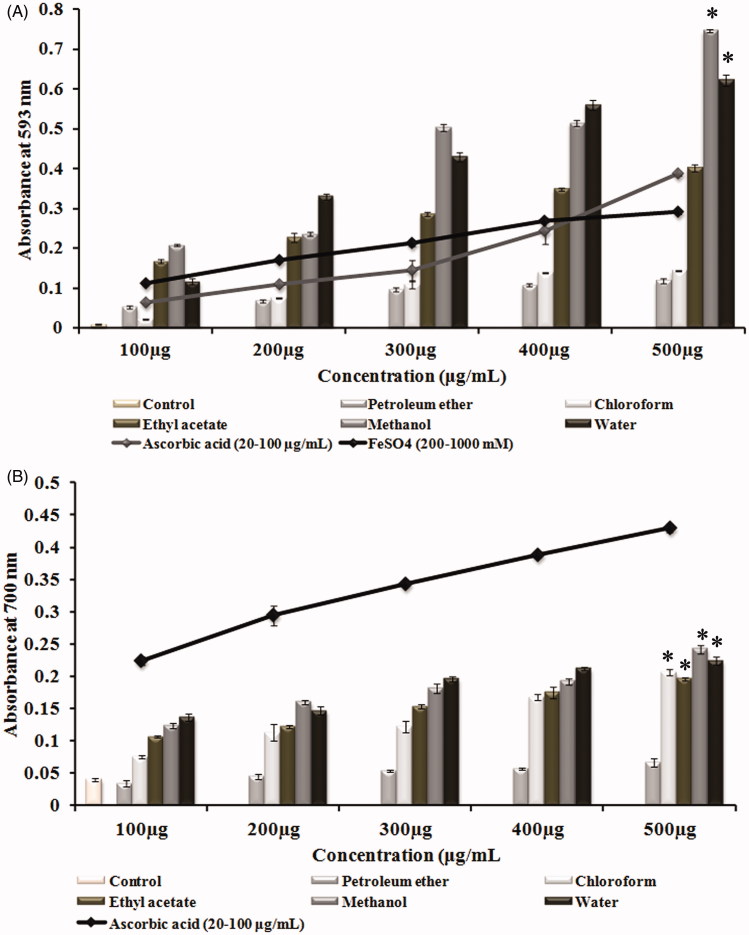
(A) Total antioxidative power of different solvent extracts of *G. tiliaefolia* (100–500 μg/mL) in comparison with standard ascorbic acid (20–100 μg/mL). (B) Reducing power of different solvent extracts of *G. tiliaefolia* (100–500 μg/mL) and standard ascorbic acid (20–100 μg/mL). *Significant level at *p* < 0.05 (control versus treated).

Antioxidative power as explained by FRAP assay describes the electron-donating ability of various solvent extracts of *G. tiliaefolia*. FRAP values are expressed in mM equivalent of Fe(II)/L. The results showed that all the extracts of *G. tiliaefolia* even at 100 μg/mL exhibited significant ferric reducing capacity, of which, methanol extract showed the highest with the absorbance of 0.208 ± 0.002, that is equivalent to the absorbance of 600 mM/L of Fe(II) in FeSO_4_ in comparison with positive control ascorbic acid (80 μg/mL) (Figure 2(A)). Results of reducing power assay, which is an indicator for the reduction of Fe^3+ ^to Fe^2+ ^, also substantiate with the FRAP results. Methanol extract was found to have a relatively higher reduction potential with the highest absorbance of 0.242 ± 0.007 (500 μg/mL), when compared with other extracts and control (Figure 2(B)). Quantification of total phenolics illustrates (Table 1) that the polar solvent extracts like ethyl acetate, methanol and water extracts have higher phenolic content which correlates with the highest antioxidant capacity.

**Table 1.** Total polyphenolic content of various solvent extracts of *G. tiliaefolia* leaf.

Solvent extract	Total polyphenolic content (μg of GAE/mg of extract)
Petroleum ether	64.03 ± 9.4
Chloroform	252.9 ± 9.4
Ethyl acetate	249.8 ± 7
Methanol	275.7 ± 7.5
Water	228.4 ± 15.1

### Cholinesterase inhibitory assay

**Figure 3.**
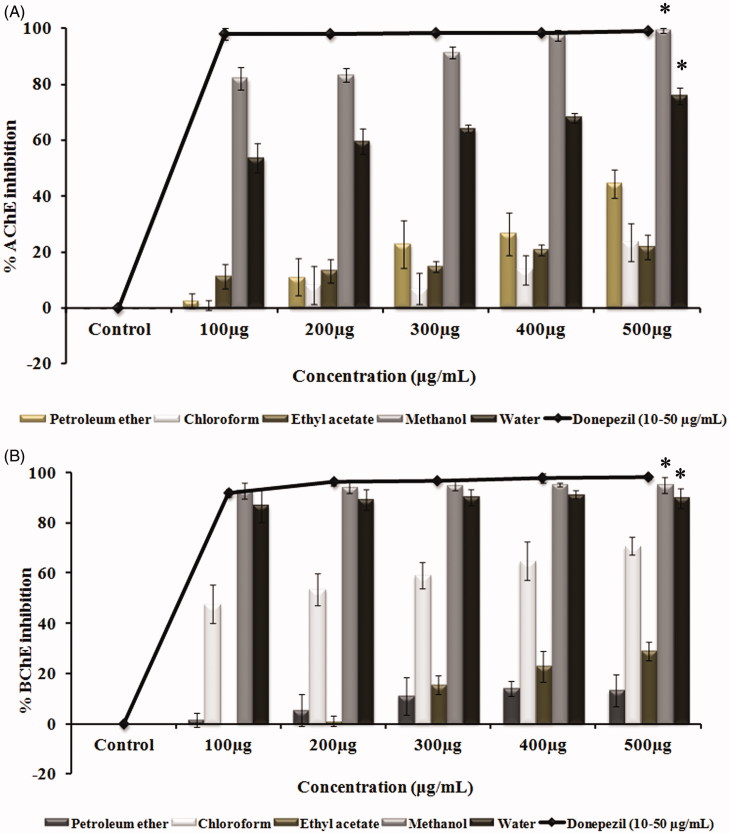
(A) Acetylcholinesterase inhibitory effect and (B) butyrylcholinesterase inhibitory effect of various solvent extracts (100–500 μg/mL) of *G. tiliaefolia* and standard donepezil (10–50 μg/mL). *Significant level at *p* < 0.05 (control versus treated).

The study also reveals the role of *G. tiliaefolia* as a dual cholinesterase inhibitor. The inhibition of cholinesterase enzymes by the leaf extracts of *G. tiliaefolia* showed that the polar solvent extracts (methanol and water) showed higher inhibition when compared with that of non-polar solvent extracts. The highest cholinesterase inhibitory activity was found to be in methanol and water extracts with IC_50_ values of 60.9 ± 1.1 and 94.7 ± 6.3 μg/mL for AChE (Figure 3(A)) and 53.7 ± 0.7 and 63.04 ± 4.4 μg/mL for BChE (Figure 3(B)), respectively. The results indicate the effectiveness of methanol extract in impeding the role of cholinesterase enzyme in hydrolyzing the neurotransmitter ACh.

### Anti-aggregation assay

**Figure 4.**
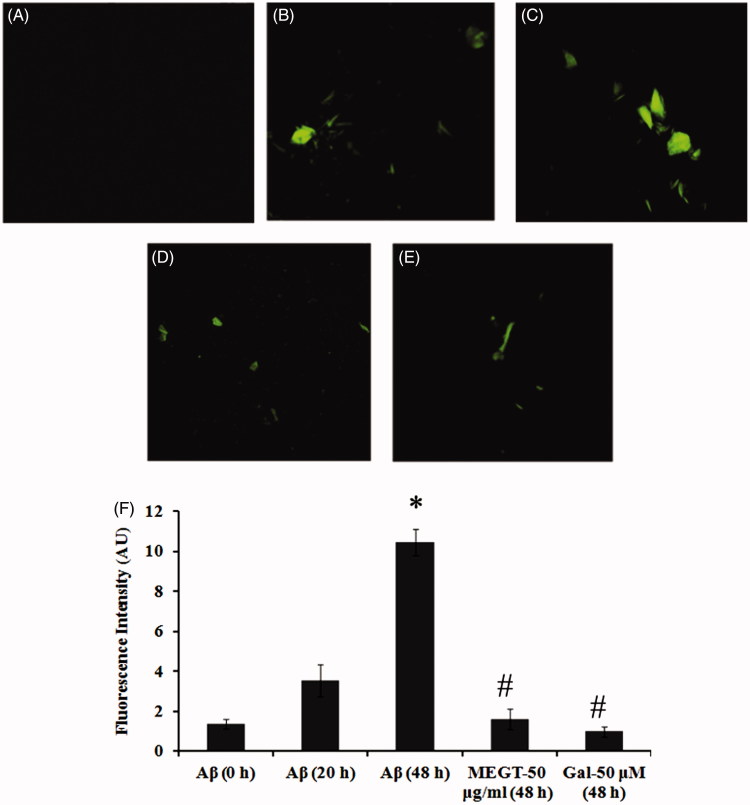
Confocal microscopic images indicating the anti-aggregation effect of MEGT (A) Aβ_25–35_ (0 h), (B) Aβ_25–35_ (20 h), (C) Aβ_25–35_ (48 h), (D) MEGT-50 μg/mL (48 h) and (E) galanamine-50 μM (48 h). (F) Quantification of anti-aggregation of Aβ_25–35_ by MEGT by Th-T fluorimetric assay. Significant level at *p* < 0.05 (*Aβ_25–35_ (0 h) versus Aβ_25–35_ (48 h); #Aβ_25–35_ (48 h) versus GT-50 μg/mL (48 h)/Gal-50 μM (48 h)).

**Figure 5.**
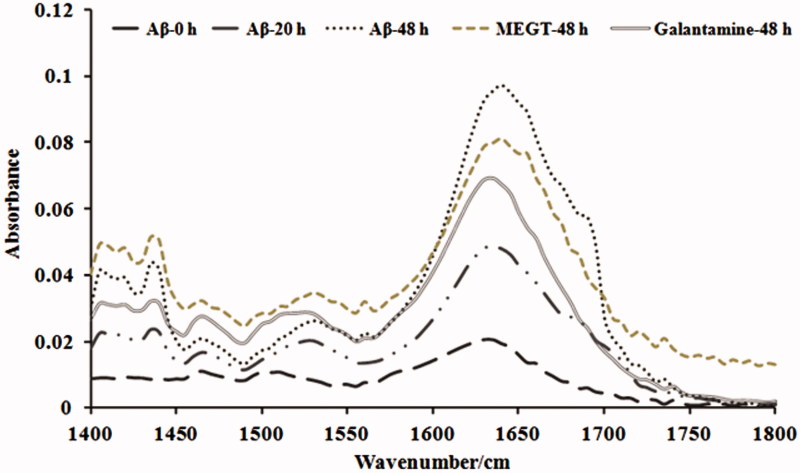
FTIR analysis indicating the anti-aggregation effect of MEGT against Aβ_25–35_.

Since the preliminary experiments showed methanol extract to be effective, we studied the effect of methanol extract in inhibiting the aggregation of Aβ_25–35_ by Th-T assay, confocal microscopic and FTIR analysis. In Th-T assay, an increase in fluorescence intensity was observed in the Aβ_25–35_ group indicating the formation of oligomers and aggregates as time increases (0–20 and 48 h). However, treatment with methanol extract of *G. tiliaefolia* (50 μg/mL) significantly (*p* < 0.05) reduced the fluorescence intensity similar to that of galantamine (50 μM) (Figure 4(F)). The results were supported by the evidence from confocal microscopic analysis, where it shows reduced fibril formation upon MEGT treatment (Figure 4(A–E)). The results indicate that *G. tiliaefolia* extract has the ability to decrease the fibril formation by halting the self-assembly of Aβ_25–35_ and prevent oligomerization. Further, FTIR analysis was performed to check the secondary structure formation in the presence and absence of MEGT. A sharp increase in peak intensities in the amide I spectral region 1600–1700 cm^−1^ was obtained in the Aβ_25–35_ group in a time-dependent manner (0–48 h) (Figure 5). However, co-incubation with MEGT (50 μg/mL) and galantamine treatment reduced the peak intensity indicating the prevention of aggregation. The results positively confirm the anti-aggregation property of MEGT.

### *Grewia tiliaefolia* pretreatment prevented Aβ_25–35_ induced toxicity in Neuro2a cells

**Figure 6.**
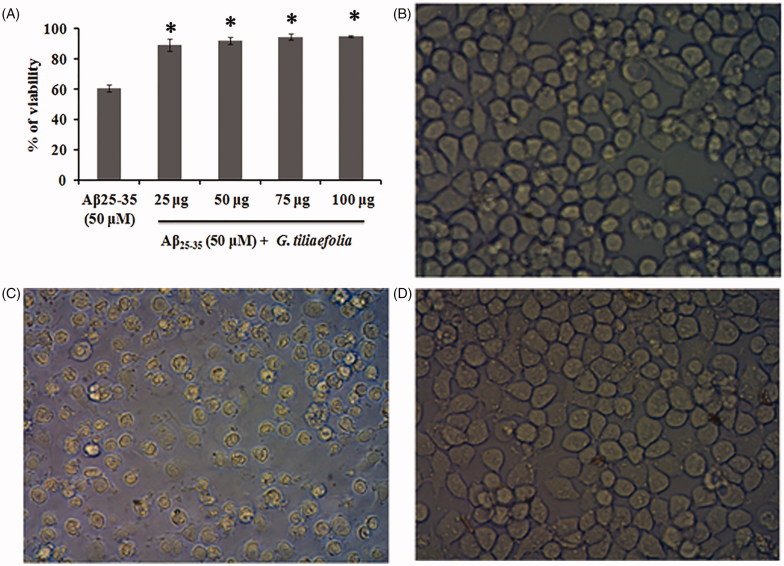
(A) Protective effect of methanol extract of *G. tiliaefolia* against Aβ_25–35_-induced toxicity in Neuro2A cells. *Significant level at *p* < 0.05 (Aβ_25–35_ treated versus *G. tiliaefolia* treated). Microscopic images depicting the protective effect of *G. tiliaefolia* against Aβ_25–35_ toxicity, (B) control, (C) Aβ_25–35_ treated and (D) Aβ_25–35 _+ methanol extract of *G. tiliaefolia* treated.

Treatment of Aβ_25–35_ to the neuro-2A cells for 24 h significantly reduced the cell viability (40% reduction). However, pretreatment with methanol extract of *G. tiliaefolia* (25–100 μg/mL) resulted in the reduction of Aβ_25–35_ induced cytotoxicity and significantly restored the viability of the cells (Figure 6(A)). The increase in cell viability may be due to the high anti-oxidant potential of the extract which could have minimized the oxidative stress occurred due to Aβ_25–35_ toxicity. The reduction in cell viability upon Aβ_25–35_ treatment and the restoration of viability upon extract pretreatment are shown in Figure 6(B—D).

### Bioactive guided fractionation and identification of compounds

**Figure 7.**
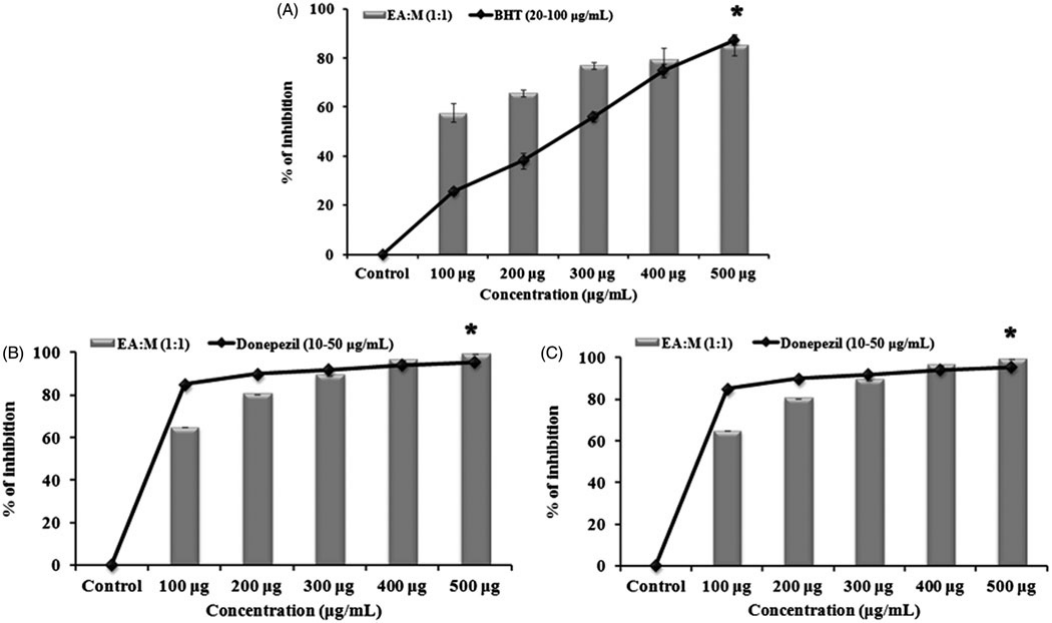
Free radical and cholinesterase inhibition of F-12 eluted with ethyl acetate:methanol (1:1): (A) DPPH scavenging activity, (B) acetylcholinesterase inhibitory effect and (C) inhibitory effect of F12 (100–500 μg/mL). Significant level at *p* < 0.05 (control versus treated).

Separation of compounds present in methanol extract through column chromatography resulted in the elution of 13 fractions of which F-12 eluted with ethyl acetate:methanol (1:1) showed the highest inhibitory activity against AChE (99%) and BChE (98%). The free radical scavenging activity of this fraction as described by DPPH assay showed 85.3% inhibition of free radical production when compared with the control. The observed results are shown in Figure 7(A–C). Since F-12 showed significant inhibition of free radical and cholinesterase enzyme, it was further subjected to LC-MS analysis and Table 2 depicts the list of compounds identified through it.

**Table 2.** Compounds identified in F-12 through LC-MS analysis.

S. no	Compound name	Molecular mass
1	Betulin	442.73
2	Lupeol	426.73
3	Harman	182.23
4	Nitidanin	404.42
5	Vitexin	432.39
6	Isovitexin	432.38
7	Squalene	410.73
8	Cyclobuxine	386.63
9	β-Amyrin	426.74
10	Betulinic aldehyde	440.71
11	Vitexin-4-*O*-glucoside	594.53
12	3-Methyl ellagic acid	316.22
13	Ergotamine tartrate	656.72
14	Adenine	135.13
15	Valeric acid	102.14

### Molecular docking

**Figure 8.**
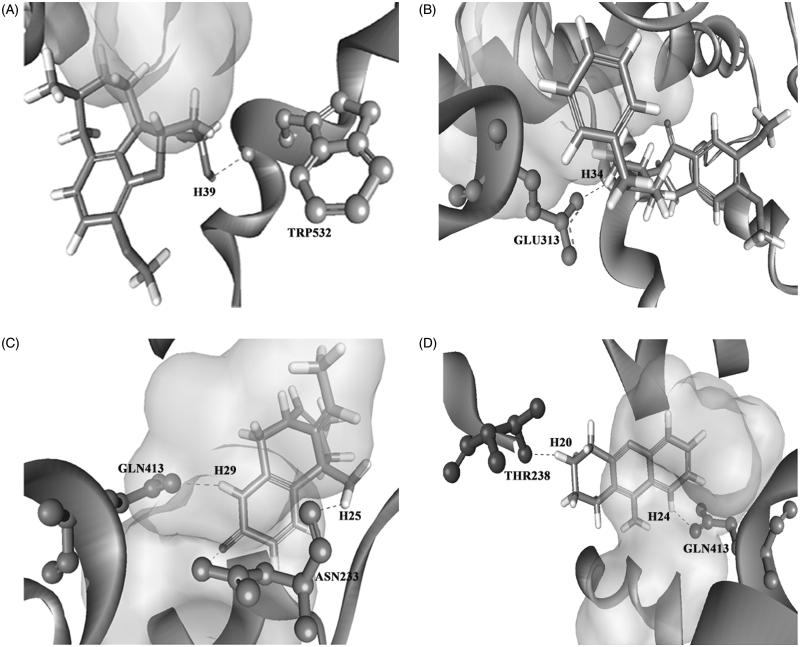
Binding mode of the positive controls: (A) galantamine, (B) donepezil, (C) huperzine A and (D) tacrine on the target site of AChE.

**Figure 9.**
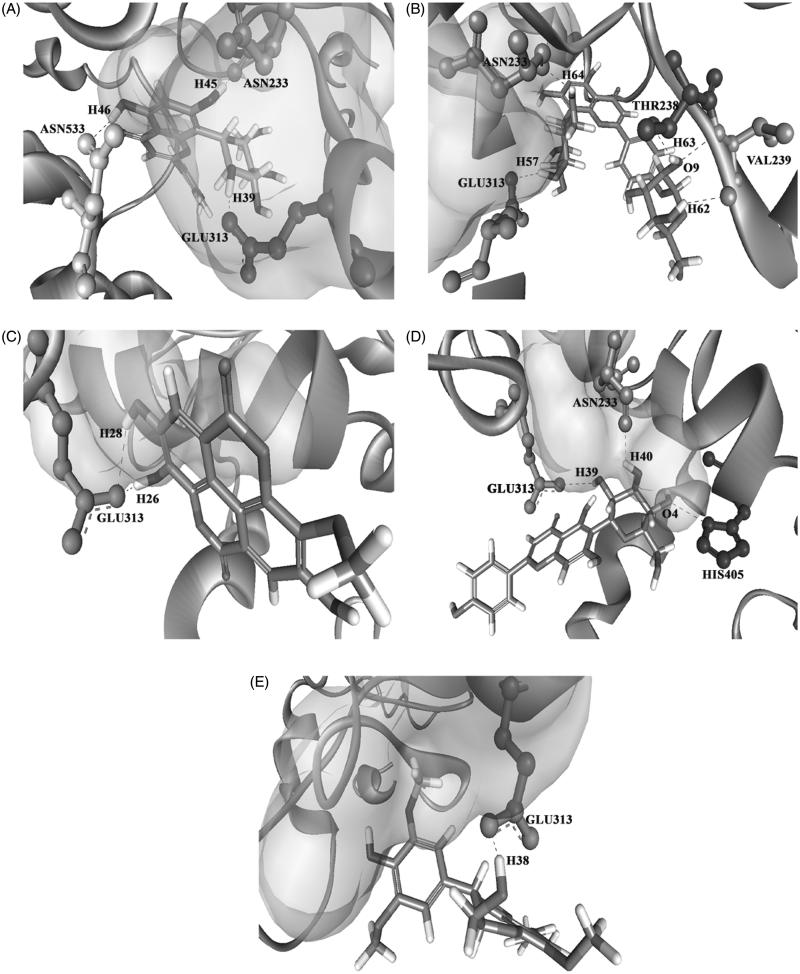
3D structure of the docked tested ligands against AChE vitexin (A), vitexin-4-*O*-glucoside (B), 3-*O*-methyl ellagic acid (C), isovitexin (D) and nitidanin (E).

Before proceeding with all the identified compounds, we performed molecular docking analysis to scrutinize the compounds which have the ability to bind to the target site and compared their efficacy with that of positive controls. Binding of the identified compounds from LC-MS analysis to the AChE is shown in Table 3. ASN233, THR238, GLU313 and TRP532 are identified as the active site residues that are essential for establishing strong interaction for the positive control compounds (donepezil, galantamine, huperzine A and tacrine) with the AChE. All the test compounds have shown a similar kind of hydrogen bond formation with the residues ASN233, THR238, GLU313 and TRP532 in AChE except β-amyrin which showed hydrogen bond interaction with VAL239. From the dock score analysis, vitexin was observed with a highest dock score of 68.105 indicating strong receptor–ligand interactions. The higher the dock score, the higher the binding efficiency between the enzyme and the ligand. Vitexin significantly established interactions with ASN233 and GLU313 residues of the AChE through O–H bonding, which is followed by vitexin-4-*O*-glucoside, 3-*O*-methyl ellagic acid, isovitexin and nitidanin with the dock scores of 59.375, 53.811, 47.606 and 45.529, respectively. The interaction of the positive controls and the top five hits of the tested ligands against AChE enzyme is shown in Figures 8 and 9. The interaction of these compounds with the target site may be the reason for the observed cholinesterase inhibition of the *G. tiliaefolia* extract.

**Table 3.** Tested ligands with their docking score and their interactions with the AChE receptor sites.

S. no.	Compound name	Pubchem ID	Docking score	Donor	Acceptor	Distance
*Positive ligands*						
1	Galantamine	9651	45.363	H39	TRP532	2.463
2	Donepezil	3152	31.04	H34	GLU313	1.937
3	Huperzine A	44461111	18.386	H25	ASN233	2.467
				H29	GLN413	2.286
4	Tacrine	1935	2.552	H20	THR238	2.046
				H24	GLN413	1.938
*Test ligands*						
1	Vitexin	5280441	68.105	H39	GLU313	1.138
				H45	ASN233	1.230
				H46	ASN533	1.941
2	Vitexin-4-*O*-glucoside	44257745	59.375	H57	GLU313	1.837
				H62	VAL239	2.412
				H63	THR238	2.367
				H64	ASN233	1.981
3	3-*O*-Methyl ellagic acid	13915428	53.811	H26	GLU313	1.052
				H28	GLU313	2.464
4	Isovitexin	162350	47.606	H39	GLU313	2.461
				H40	ASN233	2.032
				HIS405(N)	O4	2.748
5	Nitidanin	11188887	45.529	H38	GLU313	1.309
6	Betulin	72326	44.047	H62	THR238	2.070
				H76	THR238	1.895
7	Lanosterol	246983	42.744	H72	GLU313	1.477
8	Cyclobuxine	260437	40.296	H59	TRP532	2.463
9	Betulin aldehyde	99615	39.215	H67	ASN233	2.038
10	Cycloartenol	92110	35.455	H67	GLU313	1.092
11	Squalene	1105	34.896	H66	THR238	2.103
12	Valeric acid	7991	28.995	H17	GLU313	1.059
13	Lupeol	259846	25.641	H58	ASN233	2.036
14	β-Amyrin	73145	21.501	H81	VAL239	1.903

### Antioxidant and cholinesterase inhibitory activity of vitexin

**Figure 10.**
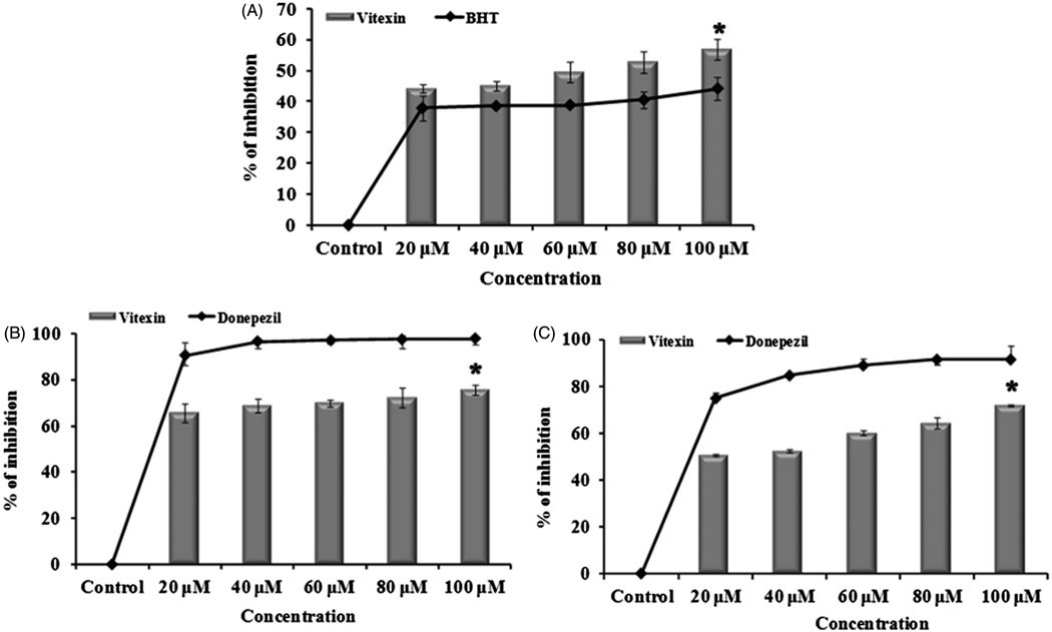
(A) Free radical scavenging assay of vitexin and standard BHT (20–100 μM) (A). (B) Acetylcholinesterase and (C) butyrylcholinesterase inhibitory effect of vitexin (20–100 μM) and the reference donepezil. *Significant level at *p* < 0.05 (control versus treated).

**Figure 11.**
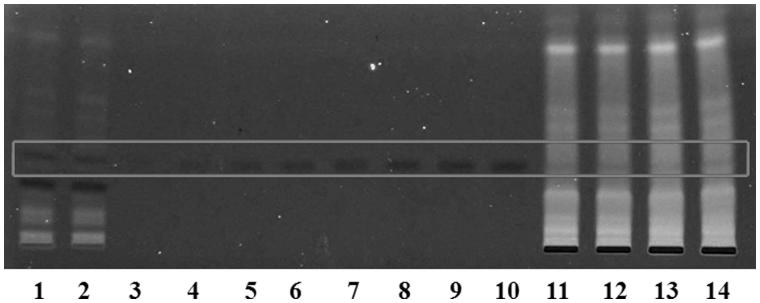
HPTLC quantification of vitexin in the extract and column fraction. (i) Lanes (1 and 2) – methanol extract, (ii) lanes (3–10) – standard vitexin and (iii) lanes 11–14 – fraction-12.

The result of *in silico* work has been validated with the *in vitro* studies where vitexin showed potent dual cholinesterase inhibition. The results of antioxidant and cholinesterase inhibitory effect of vitexin are represented in Figure 10(A–C). The activity of vitexin was dose dependent and, at 100 μM concentration, it showed 75.7 ± 2.24% and 71.7 ± 0.5% (significant inhibition, *p* < 0.05) for both AChE and BChE, respectively. Radical scavenging activity of vitexin as assessed by DPPH assay also showed significant (*p* < 0.05) antioxidant property when compared with the control with the IC_50_ value of 85.11 ± 7.02 μM. To further confirm the presence of vitexin, HPTLC analysis was performed, and it was found that the methanol extract contains 4.04 mg vitexin/g of extract (Figure 11).

## Discussion

Medicinal plants, since ancient times, have been used by all ethnic groups as a source of medicine. Attention towards medicinal plants as a re-emerging treatment strategy has gained importance because of the increase in the cost of synthetic medicines and their adverse effects. The present study reveals the neuroprotective effect of the medicinal plant *G. tiliaefolia* commonly found in Western and Eastern Ghats of India.

It is well established that defects in cholinergic function coupled with severe oxidative stress can play an important role in the aetiology of AD. Oxidative stress causes disruption in the cellular homeostasis by causing DNA oxidation, lipid peroxidation and protein oxidation (Butterfield & Lauderback 2002). Also, the high nitroxidative stress can initiate a cascade of redox reactions which can trigger apoptosis and evoke cytotoxic effects on neurons and endothelial cells (Estévez & Jordán 2002; Malinski 2007). One of the treatment strategies to cease the production of these free radicals is by the use of strong antioxidants. So, any compound or formulation which can act as an antioxidant as well as cholinesterase inhibitor may prove to be a better drug option for the treatment of AD, where oxidative stress and cholinesterase mechanism are involved. In the present study, methanol extract of *G. tiliaefolia* leaf has shown significant free radical scavenging effect and high reducing power, which proves that this extract has rich source of antioxidants. Compounds with reducing power were reported to hamper the lipid peroxidation caused by free radicals by acting as an electron donor and protects the cells from injury (Chanda & Dave 2009).

The study also reveals the role of *G. tiliaefolia* as a dual cholinesterase inhibitor. The acetyl and butyryl cholinesterases are the biologically important enzymes that help to hydrolyze the neurotransmitter acetylcholine. A decrease in the cholinergic activity and a disruption of synaptic function are mainly responsible for the memory impairment in AD (Sayer et al. 2004). One of the therapeutic approaches for the improvement of cognitive function in patients with AD is to potentiate the cholinergic function in the brain by the use of cholinesterase inhibitors. Cholinesterase inhibitors prolong the existence of acetylcholine released into the synaptic cleft and improve the cholinergic function. Also, they reduce the accumulation of toxic amyloid beta aggregates, the prime toxic substance in AD (Verhoeff, 2005). The cholinesterase inhibitory activity of methanol extract of *G. tiliaefolia* indicates the potency of the plant to be considered for the identification of compounds for the treatment of AD.

Aggregation and deposition of toxic Aβ in the brain cause severe neuronal damage in AD. One of the therapeutic interventions is the use of molecules that inhibit the aggregation of Aβ. Aβ_25–35_ is the shortest fragment obtained by the proteolytic processing of secretases and mimics the behaviour, aggregation pattern and toxicity of the full-length peptide (Clementi et al. 2005). Results of Th-T assay, confocal microscopy and FTIR analysis show that methanol extract of *G. tiliaefolia* effectively inhibited the aggregate formation confining its role not only to cholinesterase inhibition but also multi-targeting. The present study also points out to the neuroprotective effect of methanol extract of *G. tiliaefolia* against Aβ_25–35_-induced neurotoxicity in Neuro2A cells. As reported by several research groups, Aβ_25–35_ caused cell death, which could be due to the generation of oxidative stress upon Aβ_25–35_ treatment. The protective effect observed by methanol extract of *G. tiliaefolia* can be attributed to its high antioxidant activity as well as the anti-aggregation activity, which could have prevented ROS formation and interacted with the Aβ_25–35_ peptide preventing its oligomerization thereby mitigating the toxicity.

The identification of compounds was done by LC-MS analysis. Most of the compounds reported in the active fraction F12 were found to be mild to strong antioxidants which can ameliorate oxidative stress and enhance memory. Vitexin was identified to reverse scopolamine-induced memory dysfunction in rats (Abbasi et al. 2013). Lupeol was shown to protect mouse hippocampal cell lines HT-22 against glutamate toxicity (Brimson et al. 2012). Ellagic acid was reported to exhibit neuroprotective effects against oxidative damage in streptozotocin-induced diabetic rats (Uzar et al. 2012). Harman isolated from coffee was identified as a potent inhibitor of monoamino oxidase which is a well-known target for antidepressant and neuroprotective drugs (Herraiz & Chaparro 2006).

Computational docking of test ligands to a biological target to study their ability to interact in all possible poses in the binding pocket has become an approach in the screening of possible drug candidates against the target (Mohan et al. 2005). *In silico* docking analysis of compounds obtained by LC-MS analysis against AChE enzyme showed efficient hydrogen binding with the high docking score indicating that these compounds can bind strongly to AChE than that of the standard drugs donepezil, galantamine, huperzine A and tacrine. The standard drugs showed interaction with the binding site residues GLU313 (donepezil), TRP532 (galantamine), ASN233 (huperzine A), THR238 (tacrine) and GLN413 (huperzine A and tacrine). Most of the tested ligands showed hydrogen bond interaction with the GLU313 residue similar to those of donepezil. The interaction of these compounds with the target site may be the reason for the observed cholinesterase inhibition of the *G. tiliaefolia* extract. The result of *in silico* work has been well supported by the *in vitro* studies where the top hit vitexin showed potent dual cholinesterase inhibition. The neuroprotective effect exerted by *G. tiliaefolia* can be attributed to the presence of several biologically active phytoconstituents.

Further studies will provide sufficient insight into the molecular mechanism targeted by the plant in exerting neuroprotection.

## Conclusion

In conclusion, our study was the first to document the anti-cholinesterase activity of *G. tiliaefolia* and these findings reveal for the first time the function of traditionally important medicinal plant *G. tiliaefolia* to be utilized in the treatment of AD. Also, from our investigation, vitexin can be considered as a promising molecule in the therapeutic intervention of AD.
